# Supplementary material for: Multipotent neural stem cells originating from neuroepithelium exist outside the mouse central nervous system
Source: Nat Cell Biol. 2025 Apr 10;27(4):605–18. doi: 10.1038/s41556-025-01641-w (PMC11991921; doi:10.1038/s41556-025-01641-w)
Supplement: Supplementary file 1 — Supplementary Figs. 1 and 2 and respective legends. [file 41556_2025_1641_MOESM1_ESM.pdf]

nature cell biology

Supplementary information https://doi.org/10.1038/s41556-025-01641-w

Multipotent neural stem cells originating from neuroepithelium exist outside the mouse central nervous system

In the format provided by the authors and unedited

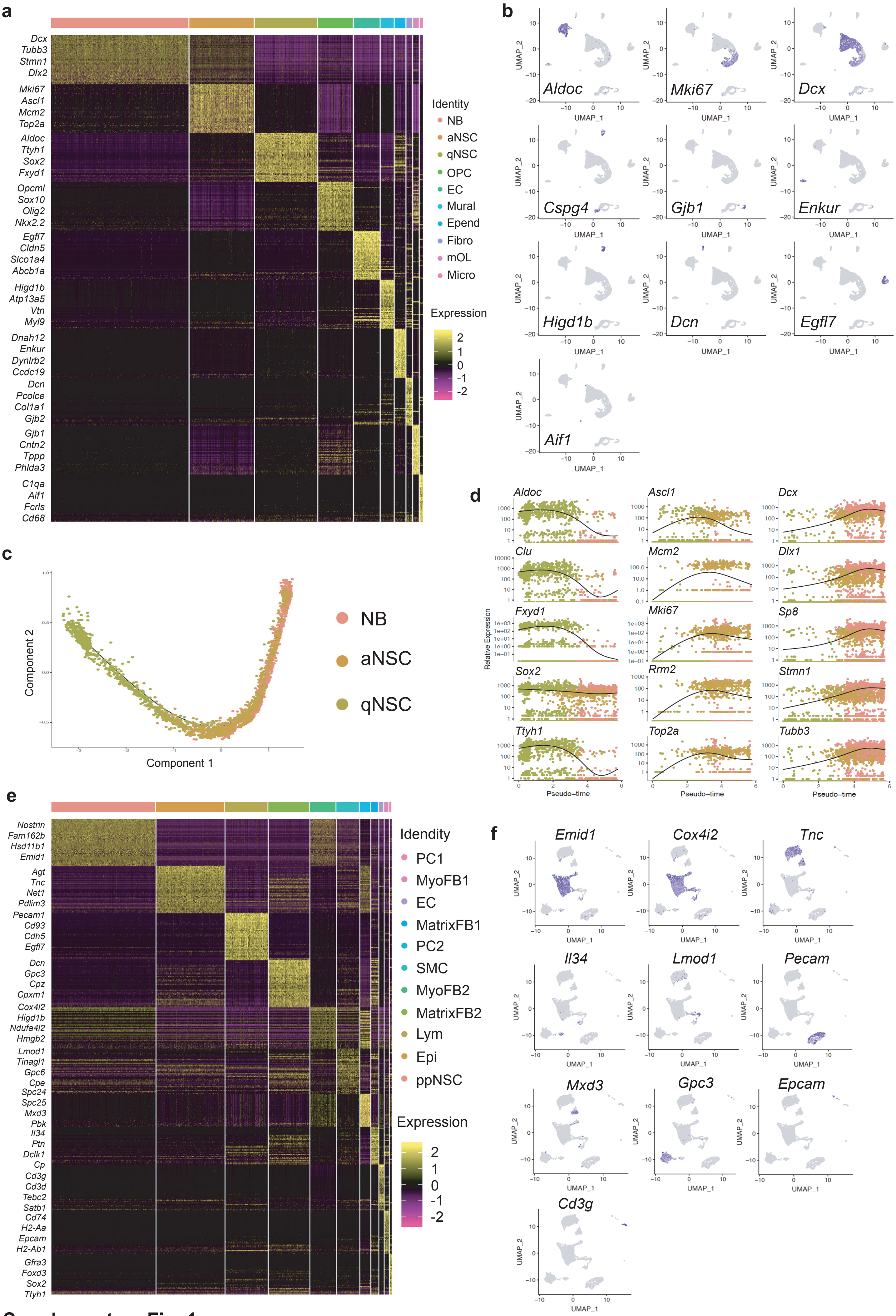

Supplementary Fig. 1

Supplementary Fig. 1 Single-cell RNA-seq analysis of brain and lung Nes-GFP^{+} cells.
a, Heatmap of the expression of the top 50 marker genes in different brain populations. Each column represents a cell and each row represents a gene. **b**, UMAP showing the expression of representative markers for each population in brain Nes-GFP^{+} cells. **c**, Pseudotemporal ordering of brain qNSC, aNSC and NB cells. **d**, Expression profile of representative genes along the pseudotime. The y axis depicts gene expression as transcript count and the running mean is in black. Each dot represents a cell and cells are highlighted in the same color gradient representing the same clusters in (**c**). **e**, Heatmap of the expression of the top 50 marker genes in different lung populations. **f**, UMAP showing the expression of representative markers for each population in lung GFP^{+} cells.

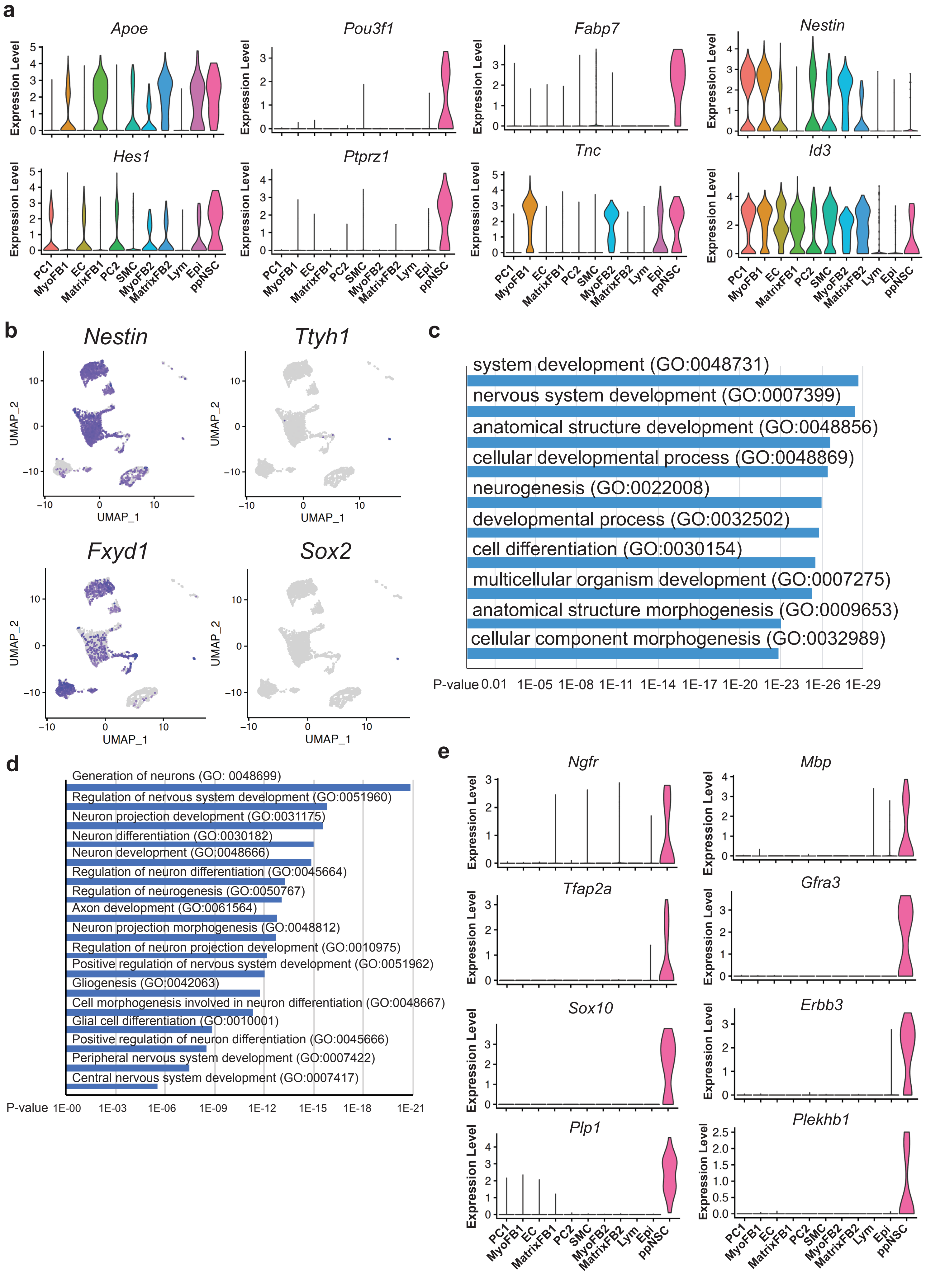

Supplementary Fig. 2

Supplementary Fig. 2 Analysis of Nes-GFP$^+$ lung neural cell cluster.

a, Violin plots showing the expression of NSC marker genes in different populations of lung tissue. **b**, UMAP showing the expression of NSC marker genes in different populations of lung tissue. **c**, Top 10 Gene Ontology (GO) terms of neural cell cluster-expressing genes. P value calculated by FISHER test. **d**, Representative Gene Ontology terms of neural cell cluster of lung tissue. P value calculated by FISHER test. **e**, Violin plots showing the expression of Schwann cell marker genes in different populations of lung tissue.
