# Supplementary material for: Multipotent neural stem cells originating from neuroepithelium exist outside the mouse central nervous system
Source: Nat Cell Biol. 2025 Apr 10;27(4):605–18. doi: 10.1038/s41556-025-01641-w (PMC11991921; doi:10.1038/s41556-025-01641-w)
Supplement: Supplementary file 2 — Reporting Summary [file 41556_2025_1641_MOESM2_ESM.pdf]

nature research

Corresponding author(s): Hans R. Schöler

Last updated by author(s): Jan 16, 2025

Reporting Summary

Nature Research wishes to improve the reproducibility of the work that we publish. This form provides structure for consistency and transparency in reporting. For further information on Nature Research policies, see our Editorial Policies and the Editorial Policy Checklist.

Statistics

For all statistical analyses, confirm that the following items are present in the figure legend, table legend, main text, or Methods section.

n/a	Confirmed	
☐	☒	The exact sample size (n) for each experimental group/condition, given as a discrete number and unit of measurement
☐	☒	A statement on whether measurements were taken from distinct samples or whether the same sample was measured repeatedly
☒	☐	The statistical test(s) used AND whether they are one- or two-sided<br>*Only common tests should be described solely by name; describe more complex techniques in the Methods section.*
☒	☐	A description of all covariates tested
☒	☐	A description of any assumptions or corrections, such as tests of normality and adjustment for multiple comparisons
☐	☒	A full description of the statistical parameters including central tendency (e.g. means) or other basic estimates (e.g. regression coefficient) AND variation (e.g. standard deviation) or associated estimates of uncertainty (e.g. confidence intervals)
☐	☒	For null hypothesis testing, the test statistic (e.g. F, t, r) with confidence intervals, effect sizes, degrees of freedom and P value noted<br>*Give P values as exact values whenever suitable.*
☒	☐	For Bayesian analysis, information on the choice of priors and Markov chain Monte Carlo settings
☒	☐	For hierarchical and complex designs, identification of the appropriate level for tests and full reporting of outcomes
☒	☐	Estimates of effect sizes (e.g. Cohen's d, Pearson's r), indicating how they were calculated

Our web collection on statistics for biologists contains articles on many of the points above.

Software and code

Policy information about availability of computer code

Data collection	Applied Biosystems 7500; Leica DMI6000B inverted fluorescence microscope equipped with a Hamamatsu Orca-R2 charge-coupled device camerafor capturing immunoflourescence images; Zeiss LSM 710 confocal microscope; Zeiss LSM 780 confocal microscope; FACS Canto (BD Biosciences) or FACS Aria II (BD Biosciences); Chromium (10X Genomics); BD Rhapsody Express system (BD); Illumina NextSeq500.
Data analysis	iScan (v 1.3), BeadStudio (v 3.2), LAS AF (v.3.2.0), ZEN (3.9) software, Adobe Photoshop, ImageJ (2.1.0/1.53j), Microsoft Excel, UMI-tools (version1.0.1), STAR (version 2.7.1a), Subread featureCounts (version 1.6.4), Seurat (version 3.1.5), BD Rhapsody WTA Analysis pipeline (version 1.11), Monocle (version 2.14), PANTHER (17.0), eBD FACS Diva (8.0.1), FlowJo software (10.6.1), Molecular Signatures Database (MSigDB) (3.0), MATLAB® (MathWorks®) software (R2020b) , BeadStudio (3.2) (Illumina), QUMA, PatchMaster (2.4) software, Patcher's Power Tool routine for IgorPro (WaveMetrics, Lake Oswego, OR, USA) and SciDAVis program (http://scidavis.sourceforge.net/index.html), Molecular Signatures Database (MSigDB) (3.0),eBD FACS Diva (8.01), HISAT2 (v2.2.1), Cufflinks (v.2.2.1), HTSeq (htseq-count v1.99.2), lumiExpresso (2.24.0), R-Bioconductor (3.0).

For manuscripts utilizing custom algorithms or software that are central to the research but not yet described in published literature, software must be made available to editors and reviewers. We strongly encourage code deposition in a community repository (e.g. GitHub). See the Nature Research guidelines for submitting code & software for further information.

Data

Policy information about availability of data

All manuscripts must include a data availability statement. This statement should provide the following information, where applicable:

- Accession codes, unique identifiers, or web links for publicly available datasets
- A list of figures that have associated raw data
- A description of any restrictions on data availability

The accession numbers for the microarray, bulk RNA-seq, and single-cell RNA-seq data in this study are available from Gene Expression Omnibus under accession number GEO: GSE151649, GSE213158, GSE213133. The publicly available datasets used in this study are available from Gene Expression Omnibus under accession number GEO: GSE30500, GEO3781644 and GEO6499593. The mouse reference genome for RNA-seq analysis is GRCm38 (https://cloud.biohpc.swmed.edu/index.php/s/grcm38_tran/download). Source data are provided with this study. All other data supporting the findings of this study are available from the corresponding author on reasonable request.

Field-specific reporting

Please select the one below that is the best fit for your research. If you are not sure, read the appropriate sections before making your selection.

☒ Life sciences ☐ Behavioural & social sciences ☐ Ecological, evolutionary & environmental sciences

For a reference copy of the document with all sections, see nature.com/documents/nr-reporting-summary-flat.pdf

Life sciences study design

All studies must disclose on these points even when the disclosure is negative.

Sample size	No statistical methods were used to predetermine sample size. For each experiment, sample size was chosen based on the common methods in literatures and our previous experiences.
Data exclusions	No data were excluded from the analysis.
Replication	The majority of experiments was independently repeated at least three times with successful replication, with the exception of single cell RNA-sequencing which was performed once. The number of independent experiments was provided in the figures legends or in the method.
Randomization	No randomization was used in this study. Randomization is not applicable to cell line studies. Animal studies were observational studies, identifying cell populations in its endogenous state without any external manipulations. Positive controls were taken from the same mice. in most cases, brain NSCs from the same mice served as control for assessing pNSCs from the lung, tail, or other organs. For negative control for the tamoxifen experiments, mice were age matched with the same genotype, except without tamoxifen injection.
Blinding	Blinding was not performed in the experiments as most experiments were conducted by the same few investigators. For experiments comprising imaging analysis, laser intensity and channel parameters were uniform across all groups in the same set of experiment. All experiments were repeated independently multiple times to validate the results.

Reporting for specific materials, systems and methods

We require information from authors about some types of materials, experimental systems and methods used in many studies. Here, indicate whether each material, system or method listed is relevant to your study. If you are not sure if a list item applies to your research, read the appropriate section before selecting a response.

Materials & experimental systems

n/a	Involved in the study	
☐	☒	Antibodies
☐	☒	Eukaryotic cell lines
☒	☐	Palaeontology and archaeology
☐	☒	Animals and other organisms
☒	☐	Human research participants
☒	☐	Clinical data
☒	☐	Dual use research of concern

Methods

n/a	Involved in the study	
☒	☐	ChIP-seq
☐	☒	Flow cytometry
☒	☐	MRI-based neuroimaging

Antibodies

Antibodies used	Primary antibody for immunocytochemistry experiments: mouse anti-Nestin (Millipore, MAB353C3, 1:200), rabbit anti-Sox2 (Cell Signaling technology, #23064, 1:1000), goat anti-Sox2 (Santa Cruz, sc-17320, 1:500), rabbit anti-Olig2 (Millipore, AB9610, 1:1000), goat anti-Sox1 (R&D, AF3369, 1:200), mouse anti-Tuj1 (Sigma, T8660, 1:1000), rabbit-anti Tuj1 (Biolegend, 802001, 1:1000), rabbit

anti-MAP2 (Santa Cruz, SC-20172, 1:1000), rabbit anti-GFAP (Millipore, AB5804, 1:500), rabbit anti-S100B (Abcam, ab41548, 1:200), mouse anti-O4 (Millipore, MAB345, 1:100), rat anti-Myelin Basic Protein (MBP) (Abcam, ab7349, 1:500), rabbit anti-glutamate (Sigma, AB5018, 1:2000), rabbit anti-GABA (Millipore, ABN131, 1:500), rabbit anti-ChAT (Millipore, AB143, 1:500), mouse anti-vesicular glutamate transporter 1 (vGluT1) (Millipore, MAB5502, 1:100), rabbit anti-p75 (Sigma, AB1554, 1:500), and rabbit anti-Sox10 (Abcam, ab155279, 1:500).
Primary antibody for immunohistochemical experiments: rabbit anti-S100B (Abcam, ab41548, 1:200), chicken anti-glial fibrillary acidic protein (Millipore, AB5541, 1:1000 or 1:500), rabbit anti-Oligo2 (Millipore, AB9610, 1:400), and mouse anti-neuronal nuclei (NEUN) (Millipore, MAB377, 1:400), rat anti-Myelin Basic Protein (MBP) (Abcam, ab7349, 1:400), rabbit anti-GFAP (Thermo Fisher Scientific, RB087A1, 1:400), goat anti-tdTomato (SICGEN, AB8181-200, 1:400), chicken anti-GFP (AVES LABS, AB_2307313, 1:400), rabbit anti-RFP (Biomol, 600-401-379, 1:400), rabbit anti-Ki67 (Abcam, ab15580, 1:400) and mouse anti-Nestin (Millipore, MAB353C3, 1:200), Rabbit anti-Sox2 (Cell Signaling, #23064, 1:400), goat anti-Sox1 (R&D, AF3369, 1:400), mouse anti-Tuj1 (Sigma, T8660, 1:400), rabbit-anti Tuj1 (Biolegend, 802001, 1:400), rabbit-anti MPZ (Thermo Fisher Scientific, #PA5-37179, 1:400), rabbit anti-Synapsin 1 (Sigma, S193, 1:300), rabbit anti-GABA (Millipore, ABN131, 1:250), rabbit anti-ChAT (Millipore, AB143, 1:250), rabbit anti-TH (Santa Cruz, sc-14007, 1:250).
Alexa fluorophore-conjugated secondary antibodies (all from Invitrogen, 1:500) and DAPI/Hoechst 33342 (Invitrogen, 1:1000) were used to visualize primary antibodies and nuclei, respectively.

Validation

All primary antibodies used in this study are commercially obtained and were validated by commercial suppliers and confirmed by specific labeling of target molecules or cell types. Secondary antibodies have been tested in our experimental conditions to rule out unspecific reactivity.
Primary antibodies were validated by the supplier, with the relevant data page listed below:
Primary antibody for immunocytochemistry experiments:
mouse anti-Nestin (Millipore, MAB353C3, 1:200):
https://www.merckmillipore.com/IE/en/product/Anti-Nestin-Antibody-clone-rat-401-Cy3-conjugate,MM_NF-MAB353C3?ReferrerURL=https%3A%2F%2Fwww.google.com%2F#anchor_COA
(see CoA)
rabbit anti-Sox2 (Cell Signaling technology, #23064, 1:1000):
https://www.cellsignal.com/products/primary-antibodies/sox2-d9b8n-rabbit-mab/23064?srsltid=AfmBOorrh3BXPX5KMBOTjupSVFNlUoYdPyEChLdmwIuK7G2ow9jDi3Ra
goat anti-Sox2 (Santa Cruz, sc-17320, 1:500):
https://www.scbt.com/p/sox-2-antibody-y-17?srsltid=AfmBOoopHDzPmqmchc6xYhfigkXM5lrnTIPov4YRG_UcRg687-oUbvEo
rabbit anti-Olig2 (Millipore, AB9610, 1:1000):
https://www.merckmillipore.com/IE/en/product/Anti-Olig-2-Antibody,MM_NF-AB9610?ReferrerURL=https%3A%2F%2Fwww.google.com%2F#anchor_COA
(see CoA)
goat anti-Sox1 (R&D, AF3369, 1:200):
https://www.rndsystems.com/cn/products/human-mouse-rat-sox1-antibody_af3369#product-datasheets
mouse anti-Tuj1 (Sigma, T8660, 1:1000):
https://www.sigmaaldrich.com/HK/en/product/sigma/t8660
rabbit-anti Tuj1 (Biolegend, 802001, 1:1000):
https://www.biolegend.com/fr-ch/products/purified-anti-tubulin-beta-3-tubb3-antibody-11579?GroupID=GROUP686
rabbit anti-MAP2 (Santa Cruz, SC-20172, 1:1000):
https://www.scbt.com/p/map-2-antibody-h-300?srsltid=AfmBOop_vL9PnH5FO8Q7gkSwBeOwYLbie58_6Rl8PSy5Gbq264GCvos8
rabbit anti-GFAP (Millipore, AB5804, 1:500):
https://www.merckmillipore.com/IE/en/product/Anti-Glial-Fibrillary-Acidic-Protein-GFAP-Antibody,MM_NF-AB5804?ReferrerURL=https%3A%2F%2Fwww.google.com%2F#anchor_COA
(see CoA)
rabbit anti-S100B (Abcam, ab41548, 1:200):
https://www.abcam.com/en-us/products/primary-antibodies/s100-beta-antibody-ab41548?srsltid=AfmBOoqBQ_MvCnHNkj0Y7hJp1ttJqQaU6vKYFR8hFQszG2gnRPaLiO5n#tab=images
mouse anti-O4 (Millipore, MAB345, 1:100):
https://www.emdmillipore.com/US/en/product/Anti-O4-Antibody-clone-81,MM_NF-MAB345#anchor_COA
(see CoA)
rat anti-Myelin Basic Protein (MBP) (Abeam, ab7349, 1:500):
https://www.abcam.com/en-us/products/primary-antibodies/myelin-basic-protein-antibody-12-ab7349?srsltid=AfmBOoqqYD_Lixbv-FPAYluB7ztPpJnRMCImiRuCm_MfKUIC-V4SvfHr#tab=images
rabbit anti-glutamate (Sigma, AB5018, 1:2000):
https://www.merckmillipore.com/IE/en/product/Anti-Glutamate-No-Glutaraldehyde-Antibody,MM_NF-AB5018?ReferrerURL=https%3A%2F%2Fwww.google.com%2F#anchor_COA
(see CoA)
rabbit anti-GABA (Millipore, ABN131, 1:500):
https://www.merckmillipore.com/IE/en/product/Anti-GABA-Antibody,MM_NF-ABN131?ReferrerURL=https%3A%2F%2Fwww.google.com%2F#anchor_COA
rabbit anti-ChAT (Millipore, AB143, 1:500):
https://www.merckmillipore.com/IE/en/product/Anti-Choline-Acetyltransferase-ChAT-Antibody,MM_NF-AB143?ReferrerURL=https%3A%2F%2Fwww.google.com%2F#anchor_COA
(see CoA)
mouse anti-vesicular glutamate transporter 1 (vGluTl) (Millipore, MAB5502, 1:100):
https://www.merckmillipore.com/IE/en/product/Anti-Vesicular-Glutamate-Transporter-1-Antibody,MM_NF-MAB5502?ReferrerURL=https%3A%2F%2Fwww.google.com%2F#anchor_COA
(see CoA)
rabbit anti-p75 (Sigma, AB1554, 1:500):
https://www.merckmillipore.com/IE/en/product/Anti-Nerve-Growth-Factor-Receptor-Antibody-p75,MM_NF-AB1554?ReferrerURL=https%3A%2F%2Fwww.google.com%2F#anchor_COA
(see CoA)
rabbit anti-Sox10 (Abcam, ab155279, 1:500):

https://www.abcam.com/en-us/products/primary-antibodies/sox10-antibody-epr4007-ab155279?
srsltid=AfmBOorLqqbXSOx4lsDqPsIaLvBHpVThBIugp7os60EhzmnhAIUbT_-6#tab=images

Primary antibody for immunohistochemical experiments:
rabbit anti-S100B (Abeam, ab41548, 1:200):
https://www.abcam.com/en-us/products/primary-antibodies/s100-beta-antibody-ab41548?
srsltid=AfmBOoqBQ_MvCnHNkj0Y7hJp1ttJqQaU6vKYFR8hFQszG2gnRPaLiO5n#tab=images
chicken anti-glial fibrillary acidic protein (Millipore, AB5541, 1:1000 or 1:500):
https://www.merckmillipore.com/IE/en/product/Anti-Glial-Fibrillary-Acidic-Protein-Antibody,MM_NF-AB5541?ReferrerURL=https%
3A%2F%2Fwww.google.com%2F#anchor_COA
(see CoA)
rabbit anti-Oligo2 (Millipore, AB9610, 1:400):
https://www.merckmillipore.com/IE/en/product/Anti-Olig-2-Antibody,MM_NF-AB9610?ReferrerURL=https%3A%2F%
2Fwww.google.com%2F#anchor_COA
(see CoA)
mouse anti-neuronal nuclei (NEUN) (Millipore, MAB377, 1:400):
https://www.merckmillipore.com/IE/en/product/Anti-NeuN-Antibody-clone-A60,MM_NF-MAB377?ReferrerURL=https%3A%2F%
2Fwww.google.com%2F#anchor_COA
(see CoA)
rat anti-Myelin Basic Protein (MBP) (Abcam, ab7349, 1:400):
https://www.abcam.com/en-us/products/primary-antibodies/myelin-basic-protein-antibody-12-ab7349?srsltid=AfmBOoqqYD_Lixbv-
FPAYluB7ztPpJnRMCImiRuCm_MfKUIC-V4SvfHr#tab=images
rabbit anti-GFAP (Thermo Fisher Scientific, RB087A, 1:400):
https://www.fishersci.com/shop/products/gfap-glial-fibrillary-acidic-protein-rabbit-polyclonal-antibody-epredia/RB087A
goat anti-tdTomato (SICGEN, AB8181-200, 1:400):
https://store.sicgen.pt/catalog/product/AB8181
chicken anti-GFP (AVES LABS, AB_2307313, 1:400):
https://www.antibodiesinc.com/products/anti-green-fluorescent-protein-antibody-gfp
rabbit anti-RFP (Biomol, 600-401-379, 1:400):
https://www.biomol.com/products/antibodies/primary-antibodies/epitope-tag/anti-red-fluorescent-protein-rfp-600-401-379
rabbit anti-Ki67 (Abcam, ab15580, 1:400):
https://www.abcam.com/en-us/products/primary-antibodies/ki67-antibody-ab15580?
srsltid=AfmBOooWUA2BkT7tFHYgXg24rGmeCDt8F6Ez9ZL0EjzgipQgi6UiZ2ym#tab=images
mouse anti-Nestin (Millipore, MAB353C3, 1:200):
https://www.merckmillipore.com/IE/en/product/Anti-Nestin-Antibody-clone-rat-401-Cy3-conjugate,MM_NF-MAB353C3?
ReferrerURL=https%3A%2F%2Fwww.google.com%2F#anchor_COA
Rabbit anti-Sox2 (Cell Signaling, #23064, 1:400):
https://www.cellsignal.com/products/primary-antibodies/sox2-d9b8n-rabbit-mab/23064?
srsltid=AfmBOorrh3BXPX5KMBOTjupSVFNlUoYdPyEChLdmwIuK7G2ow9jDi3Ra
goat anti-Sox1 (R&D, AF3369, 1:400):
https://www.rndsystems.com/cn/products/human-mouse-rat-sox1-antibody_af3369#product-datasheets
mouse anti-Tuj1 (Sigma, T8660, 1:400):
https://www.sigmaaldrich.com/HK/en/product/sigma/t8660
rabbit-anti Tuj1 (Biolegend, 802001, 1:400):
https://www.biolegend.com/fr-ch/products/purified-anti-tubulin-beta-3-tubb3-antibody-11579?GroupID=GROUP686
rabbit-anti MPZ (Thermo Fisher Scientific, #PA5-37179, 1:400):
https://www.thermofisher.com/antibody/product/MPZ-Antibody-Polyclonal/PA5-37179
rabbit anti-Synapsin 1 (Sigma S193, 1:300):
https://www.sigmaaldrich.com/HK/en/product/sigma/s193#product-documentation
rabbit anti-GABA (Millipore, ABN131, 1:250):
https://www.merckmillipore.com/IE/en/product/Anti-GABA-Antibody,MM_NF-ABN131?ReferrerURL=https%3A%2F%
2Fwww.google.com%2F#anchor_COA
(see CoA)
rabbit anti-ChAT (Millipore, AB143, 1:250):
https://www.merckmillipore.com/IE/en/product/Anti-Choline-Acetyltransferase-ChAT-Antibody,MM_NF-AB143?ReferrerURL=https%
3A%2F%2Fwww.google.com%2F#anchor_COA
rabbit anti-TH (Santa Cruz, sc-14007, 1:250):
https://www.scbt.com/p/th-antibody-h-196?srsltid=AfmBOop6h8X-31yUILg0ocXkxZHuHZo50MM2vwp1gCk-euzHyWl80tx4

Eukaryotic cell lines

Policy information about cell lines

Cell line source(s)

Mouse embryonic limb NSCs, adult lung NSCs, brain NSCs from WT mice were derived in house.
Mouse adult lung, postnatal lung, tail, DRG peripheral NSCs, brain NSCs from Nestin-GFP, Sox2-GFP, Wnt1-Cre-mTmG, Sox1-GFP, Sox1-Cre-mTmG, Sox1-CreERT2-EYFP mice were derived in house. All the cell lines were derived from mice of both sex randomly. HEK293 cells were obtained from American Type Culture Collection (ATCC).

Authentication

HEK293 cells were authenticated by STR profiling from the providers. Other cell lines were authenticated in house using various methods including RT-qPCR, immunofluorescence, genotyping, karyotyping and bulk RNA-seq.

Mycoplasma contamination	HEK293 cells were tested as negative. Other cell lines were not tested for mycoplasma contamination.
Commonly misidentified lines (See ICLAC register)	No commonly misidentified cell lines were used.

Animals and other organisms

Policy information about studies involving animals; ARRIVE guidelines recommended for reporting animal research

Laboratory animals	All mice used were bred and housed at the mouse facility Max Planck Institute (MPI) in Muenster and the Centre for Comparative Medicine Research (CCMR) at The University of Hong Kong (HKU), and animal handling was in accordance with MPI or HKU CCMR animal protection guidelines. The protocols for animal handling and maintenance for this study were approved by the Landesamt für Natur, Umwelt und Verbraucherschutz Nordrhein-Westfalen under the supervision of a certified veterinarian in charge of the MPI animal facility (protocols: Az 81-02.05.50.17.014, Az 84-02.04.2016.A525, Az 81-02.04.2017.A376 and Az 84-02.05.2016.A494) and by the Government of the Hong Kong Special Administrative Region Department of Health (protocol: (23-208) in DH/HT&A/8/2/3 Pt.55). C57BL/6, CD1, B6C3F1 mice were bred in house. The background of WT mice used in this study was CD1 mice or mixtures between CD1 and C3H. Nestin-GFP mouse (Stock No: 029671), Wnt1-Cre mouse (Stock No: 007807), R26-mT/mG mouse (Stock No: 007676) were from the Jackson Laboratory and bred in house with WT mice. Sox1-GFP mice were kindly provided by Professor Stavros Malas (University of Nicosia Medical School). R26-RFP mice were kindly provided by Professor Ralf Admas (Max Placnk Institute for molecular biomedicine). Sox2-GFP mice were generated in house. Sox1-Cre sperm were got from RIKEN BRC and Sox1-Cre mice were generated in house. Sox1-CreERT2-R26-EYFP mice were kindly provided by Professor Vasso Episkopou and Professor Robin Lovell-Badge. All transgenic mice were bred with WT mice in house for experiments. Adult WT or transgenic mice with age around 8-12 weeks were used for breeding. Adult WT or transgenic mice with age around 4-6 weeks were used for experiments. Postnatal mice with age day 1-5 were used for experiments. Embryonic limb cells were derived from embryos at embryonic day 13.5. For the teratoma assay, SCID mice (2-3 months old) were used. For the in vivo transplantation experiments, NOD. Cg-Prkdcscid Il2rgtm1Wjl/SzJ mice (8 weeks) or C57BL/6 (postnatal day 1) mice were used. Mice were maintained in the animal facility with a controlled temperature of 22 °C, 40–60% humidity, a 14:10 h light:dark photoperiod, and free access to water and food.
Wild animals	This study did not involve wild animals.
Field-collected samples	This study did not involve field-collected samples.
Ethics oversight	Animal experiments and husbandry were performed according to the German Animal Welfare guidelines and approved by the Landesamt für Natur, Umwelt und Verbraucherschutz Nordrhein-Westfalen (State Agency for Nature, Environment and Consumer Protection of North Rhine-Westphalia). Animal experiments and husbandry were approved by the Government of the Hong Kong Special Administrative Region Department of Health and performed according to HKU CCMR regulations.

Note that full information on the approval of the study protocol must also be provided in the manuscript.

Flow Cytometry

Plots

Confirm that:

☒ The axis labels state the marker and fluorochrome used (e.g. CD4-FITC).

☒ The axis scales are clearly visible. Include numbers along axes only for bottom left plot of group (a 'group' is an analysis of identical markers).

☒ All plots are contour plots with outliers or pseudocolor plots.

☒ A numerical value for number of cells or percentage (with statistics) is provided.

Methodology

Sample preparation	Adult or postnatal Nestin-GFP mice, Wnt1-Cre-mTmG mice, Sox2-GFP mice, Sox1-GFP mice, Sox1-Cre-mTmG, Sox1-CreERT2-R26-EYFP mice were sacrificed, lung, brain, tail or DRG tissues were cut and minced using a pair of scissors, and incubated with 0.25% trypsin (Invitrogen) at 37°C for 10 min. After trypsinization, 3 times volume amount of MEF medium was added, and the entire suspension was pipetted up and down to dissociate the tissues. The dissociated tissues were passed through a 40-µm cell strainer (Beckon Dickinson, BD), centrifuged and resuspended in FACS medium (D-PBS without calcium and magnesium (Sigma) supplemented with 0.3% BSA (Sigma)).
Instrument	Flow cytometry was performed using either FACS Canto (BD Biosciences) or FACS Aria II (BD Biosciences).
Software	The data were analyzed using either eBD FACSDiva 8.01 (BD Biosciences) or FlowJo software 10.6.1 (Tree Star, Inc.).
Cell population abundance	After the sorting the cell population identity and abundance was verified by fluorescent microscope.
Gating strategy	Single viable cells were first selected based on forward scatter area/side scatter area and forward scatter width/forward scatter area gating to select for live cells and then sorted for GFP. For GFP+ cell sorting, brain GFP+ cells was used as positive control to select strong GFP+ cells.

☒ Tick this box to confirm that a figure exemplifying the gating strategy is provided in the Supplementary Information.
