# Supplementary figures and images for: Multipotent neural stem cells originating from neuroepithelium exist outside the mouse central nervous system
Source: Nat Cell Biol. 2025 Apr 10;27(4):605–18. doi: 10.1038/s41556-025-01641-w (PMC11991921; doi:10.1038/s41556-025-01641-w)

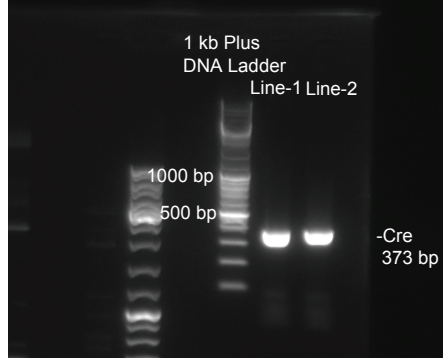

david Exp. Time: 0.28 sec Upper: 100 % Lower: 0 % Int.: 0  
Date: 22.01.2021 Time: 21:10:55

Unprocessed images of gel in Extended Data Fig. 2f

Supplement: Supplementary file 10 — Unprocessed gel image for Extended Data Fig.2f. [file 41556_2025_1641_MOESM10_ESM.pdf]
